# Exercise-Induced Skeletal Muscle Secretory Factors and Macrophage Functional Remodeling: Mechanistic Advances

**DOI:** 10.3390/ijms27177527

**Published:** 2026-08-22

**Authors:** Ziyan Li, Chenyu Lin, Linjia Tang, Yiyao Xu, Jieming Liang, Dehui Pan, Ziran Huang, Xianyan Xie, Yu Wang, Shuqi Qin, Gaoyuan Yang, Xiaoguang Liu, Huiguo Wang

**Affiliations:** School of Sport and Health, Guangzhou Sport University, Guangzhou 510500, China; 105852024100031@stu.gzsport.edu.cn (Z.L.);

**Keywords:** exercise, skeletal muscle secretory factors, myokines, macrophage functional spectrum, immunometabolism, inflammation resolution, tissue repair

## Abstract

Regular exercise mediates inter-tissue communication between skeletal muscle and the immune system through skeletal muscle-derived secretory factors, providing an important molecular basis for the beneficial effects of exercise on chronic inflammation, metabolic dysregulation, and impaired tissue repair. As key effector cells of the innate immune system, macrophages do not simply conform to a dichotomous classification of classically activated M1 macrophages and alternatively activated M2 macrophages; rather, their functional states constitute a dynamic spectrum shaped by exercise load, recovery time window, tissue microenvironment, and disease context. This review focuses on recent advances in exercise-induced skeletal muscle secretory factors involved in macrophage functional remodeling. Representative signals, including interleukin-6 (IL-6), irisin, meteorin-like protein (METRNL), fibroblast growth factor 21 (FGF21), oncostatin M (OSM), decorin, myostatin, chemokines, and extracellular vesicles, are systematically summarized in terms of their exercise responsiveness, evidence for skeletal muscle origin, and evidence supporting macrophage regulation. Based on these dimensions, an evidence-strength grading framework is further proposed. Moreover, this review integrates key signaling axes, including glycoprotein 130 (gp130)/Janus kinase (JAK)/signal transducer and activator of transcription (STAT), signal transducer and activator of transcription 6 (STAT6)/peroxisome proliferator-activated receptor gamma (PPARγ), AMP-activated protein kinase (AMPK)/nuclear factor erythroid 2-related factor 2 (Nrf2)/nuclear factor kappa B (NF-κB), transforming growth factor beta (TGF-β)/Smad, and chemokine receptor pathways, to explain how exercise-induced secretory networks participate in the dynamic regulation of the macrophage functional spectrum through immune cell recruitment, inflammatory clearance, immunometabolic reprogramming, matrix remodeling, and repair-niche formation. Current evidence indicates the translational potential of exercise-induced skeletal muscle secretory factors in skeletal muscle repair, metabolic inflammation, aging-related functional decline, and cancer rehabilitation. However, this field still faces several major challenges, including insufficient tracing of skeletal muscle-derived signals, limited direct causal validation, a lack of human tissue-level evidence, and unclear exercise dose–response relationships. Future studies should combine tissue-specific genetic interventions, receptor blockade, single-cell and spatial omics, metabolic flux analysis, and standardized human exercise interventions to further clarify the mechanistic basis and application boundaries of exercise-induced skeletal muscle–macrophage communication, thereby providing a theoretical foundation for precision exercise prescription and chronic inflammation intervention.

## 1. Introduction

Regular exercise has emerged as an important non-pharmacological strategy for preventing and managing chronic inflammatory diseases, metabolic disorders, and aging-related functional decline. Its health benefits are now understood to extend beyond traditional explanations based on energy metabolism to include endocrine regulation by skeletal muscle [1,2,3,4]. In response to exercise, skeletal muscle releases a broad repertoire of myokines and exercise-responsive secretory signals [5,6]. Among these, proteins, peptides, and cytokines produced by skeletal muscle cells and acting through autocrine, paracrine, or endocrine mechanisms are commonly referred to as myokines [6,7]. In addition, exercise can induce the release of a broader range of exercise-responsive secretory signals from skeletal muscle and other tissues, including metabolites, nucleic acids, lipid mediators, and extracellular vesicles [6,8,9]. Collectively, these secretory signals participate in inter-organ communication between skeletal muscle and adipose tissue, the liver, the vasculature, the nervous system, and the immune system, thereby providing an important molecular basis for the systemic health benefits of exercise [8,10].

Exercise-induced skeletal muscle–immune communication has become an important research direction in exercise immunology [11]. Macrophages are key effector cells of the innate immune system and are widely distributed in tissues such as skeletal muscle, adipose tissue, the liver, the lung, the intestine, and the central nervous system. They play critical roles in the initiation of inflammation, pathogen clearance, tissue repair, maintenance of metabolic homeostasis, and immune surveillance [12,13]. Owing to their high plasticity, macrophage phenotypes and functional states can dynamically change in response to cytokines, metabolic substrates, damage-associated signals, and mechanical stimuli within the local microenvironment [14,15,16]. Traditionally, macrophages have often been classified into pro-inflammatory, classically activated M1 macrophages and anti-inflammatory, reparative, alternatively activated M2 macrophages [15,17]. The former are mainly involved in inflammatory initiation, clearance of necrotic tissue, and host defense against infection, whereas the latter are closely associated with inflammation resolution, tissue remodeling, angiogenesis, and regenerative repair [18,19]. However, macrophage states in vivo do not conform to a static M1/M2 dichotomy, but instead represent a continuum of functional phenotypes characterized by contextual dependence and tissue specificity. Therefore, exercise immunology should interpret macrophage alterations from the perspectives of functional state, metabolic programming, and tissue adaptation, rather than simply classifying them as “pro-inflammatory” or “anti-inflammatory.”

The regulation of macrophage functional states by exercise is highly dependent on temporal windows and tissue microenvironments. Acute exercise may induce transient immune mobilization and tissue-adaptive responses, whereas long-term regular exercise is more closely associated with the attenuation of chronic low-grade inflammation and the improvement of metabolic homeostasis, as reflected by reductions in circulating C-reactive protein (CRP), interleukin-6 (IL-6), and tumor necrosis factor alpha (TNF-α) [11,20,21,22]. These observations suggest that exercise-mediated macrophage regulation depends on the coordinated regulation of signal transduction, immunometabolism, and tissue adaptation rather than on simple increases or decreases in individual inflammatory mediators. This framework helps explain its relevance to skeletal muscle repair, metabolic inflammation, aging-related functional decline, and cancer rehabilitation. However, the implications of macrophage functional remodeling differ substantially across disease contexts. Therefore, clarifying the distinct roles of different exercise-induced secretory signals across tissues and disease settings is essential for understanding the translational value and safety boundaries of exercise interventions.

At present, several challenges remain in this field, including unclear conceptual boundaries, uneven strength of evidence, conflation of direct and indirect effects, insufficient understanding of exercise dose–response relationships, and limited causal validation at the human tissue level. Accordingly, this review summarizes current advances in the regulation of macrophage functional states by exercise-induced skeletal muscle secretory networks, with a particular focus on the evidence hierarchy, signaling pathways, and translational implications of representative secretory signals in injury repair, metabolic inflammation, aging, and the tumor microenvironment. We emphasize that exercise does not simply suppress inflammation or unidirectionally promote M2 polarization. Rather, exercise may dynamically remodel macrophage functional states through skeletal muscle-derived secretory networks in a manner that depends on temporal windows, tissue microenvironments, and disease contexts.

## 2. Types of Exercise-Induced Skeletal Muscle Secretory Signals and the Evidence Hierarchy for Their Regulation of the Macrophage Functional Spectrum

Exercise induces a broad range of skeletal muscle secretory signals with immunomodulatory potential. Rather than driving macrophages toward a fixed phenotype, these signals shape macrophage functional states through immune-cell recruitment, inflammatory regulation, oxidative stress control, immunometabolic reprogramming, and repair-niche remodeling.

### 2.1. Chemotactic and Recruitment Factors: Establishing Local Immune Niches for Immune Cell Migration, Positioning, and Macrophage Functional Remodeling

Exercise-induced chemokines are primarily involved in immune cell mobilization, directed migration, and tissue localization. Rather than directly driving macrophages toward a fixed phenotype, these factors may shape the local distribution of immune cells and the tissue microenvironment, thereby providing the spatial and cellular basis for subsequent macrophage functional remodeling. High-intensity, prolonged, or muscle-damaging exercise increases skeletal muscle chemokine expression and promotes neutrophil, monocyte, and macrophage recruitment to exercised or injured tissue [23,24,25,26,27].

Interleukin-8 (IL-8), also known as C-X-C motif chemokine ligand 8 (CXCL8), is one of the most extensively studied representative molecules involved in exercise-induced local chemokine responses in skeletal muscle. Human studies have shown that acute exercise upregulates IL-8/CXCL8 messenger RNA (mRNA) and protein expression in skeletal muscle. This response is mainly characterized by transient local expression and paracrine activity within exercised muscle, rather than sustained elevation in the systemic circulation [28,29]. As a ligand for C-X-C motif chemokine receptor 1/2 (CXCR1/CXCR2), CXCL8 primarily recruits neutrophils and monocytes and contributes to local vascular and pre-reparative responses [29]. It therefore links contraction-induced stress to immune-cell recruitment rather than directly determining macrophage phenotype.

C-X-C motif chemokine ligand 1 (CXCL1) and C-X-C motif chemokine ligand 12 (CXCL12), also known as stromal cell-derived factor 1 (SDF-1), may also act as exercise- or injury-associated chemotactic recruitment signals involved in cell migration and tissue remodeling. CXCL1 expression can increase in serum, skeletal muscle, the liver, and other tissues after exercise, and its role appears to be more closely related to early inflammatory cell recruitment and exercise-induced stress responses [30]. CXCL12/SDF-1 primarily acts through the C-X-C motif chemokine receptor 4 (CXCR4) axis to regulate the migration of progenitor cells, myogenic cells, and vascular-associated cells, thereby contributing to skeletal muscle repair and vascular remodeling [31,32].

Although TNF-α and interleukin-1 beta (IL-1β) are not chemokines, they can shape the local inflammatory and chemokine milieu during muscle damage and repair [33,34]. Transient TNF-α signaling may promote satellite-cell activation and proliferation and support p38-dependent myogenesis [35,36], whereas sustained TNF-α exposure can inhibit myogenic differentiation through nuclear factor kappa B (NF-κB)-dependent mechanisms [33]. IL-1β may also amplify local inflammatory and chemokine signaling through the IL-6/monocyte chemoattractant protein-1 (MCP-1)/intercellular adhesion molecule 1 (ICAM-1) network, whereas prolonged exposure may inhibit myogenic differentiation [33,34].

Overall, these factors recruit and position immune cells after exercise, supporting inflammatory clearance and repair. Evidence that they directly determine terminal macrophage states remains insufficient. However, sufficient evidence is still lacking to support a direct determining role of these factors in shaping the terminal functional states of macrophages.

### 2.2. Immunoregulatory Factors: Temporal Regulation of Post-Exercise Inflammatory Responses and the Transition Toward Repair

Immunoregulatory factors are mainly involved in the temporal regulation of inflammation initiation, immune mobilization, and the transition toward repair after exercise. IL-6 is one of the most extensively studied exercise-induced myokines [37]. During sustained muscle contraction, glycogen depletion and increased metabolic stress stimulate skeletal muscle IL-6 expression and release [38,39]. Circulating IL-6 generally peaks at or shortly after exercise cessation and declines rapidly, returning to baseline within approximately 1.5–2 h in selected exercise protocols [40,41]. Its magnitude increases with exercise intensity and is amplified by low muscle glycogen, indicating a graded response rather than a universal intensity threshold [38,39]. Training may attenuate the acute plasma IL-6 response under matched absolute workloads and reduce contraction-induced muscle IL-6 mRNA expression, although the extent of attenuation remains protocol- and outcome-dependent [42,43]. Therefore, exercise-induced IL-6 should be understood as a context-dependent immunometabolic regulatory signal involved in energy mobilization and metabolic adaptation during acute exercise, rather than as a marker of the persistent pro-inflammatory activity associated with chronic inflammation.

Oncostatin M (OSM) belongs to the IL-6 cytokine family [26] and can participate in local inflammatory responses, immune cell recruitment, and tissue repair through glycoprotein 130 (gp130)-associated receptor complexes. Animal studies have shown that a single bout of aerobic exercise can upregulate OSM expression in mouse skeletal muscle, accompanied by the accumulation of neutrophils and repair-associated macrophages. Conversely, OSM deficiency attenuates these responses, supporting its involvement in post-exercise immune-cell recruitment and repair-niche remodeling [26]. This pattern is more consistent with a local, temporally regulated signal than with an abundantly circulating classical myokine.

Interleukin-15 (IL-15) is also an immunometabolism-related cytokine expressed by skeletal muscle and regulated by exercise. Its functions involve the maintenance of skeletal muscle mass, regulation of lipid metabolism, and support of natural killer (NK) cell and cluster of differentiation 8-positive (CD8+) T cells function [44,45,46]. Acute exercise can increase circulating IL-15 levels immediately after exercise [47], whereas the effects of long-term training on resting circulating IL-15 levels remain inconsistent. Current evidence is still insufficient to support a direct role of IL-15 in driving macrophage functional transitions in the context of exercise. Instead, IL-15 may indirectly influence the macrophage functional spectrum by improving the tissue metabolic environment, enhancing immune effector cell activity, and modulating local inflammatory responses.

### 2.3. Metabolic Reprogramming-Associated Exercise-Responsive Factors: Regulatory Signals in the Immunometabolic Microenvironment

Metabolic reprogramming-associated exercise-responsive factors constitute an important molecular basis linking exercise adaptation, energy metabolism, and macrophage functional remodeling. Macrophage functional states are closely coupled to their metabolic programs. Pro-inflammatory macrophages generally exhibit enhanced glycolysis to rapidly support the production of inflammatory mediators [48], whereas reparative macrophages rely more heavily on mitochondrial oxidative phosphorylation, fatty acid oxidation, and antioxidant responses to sustain inflammation resolution, matrix remodeling, and tissue repair [18,49].

Irisin is an exercise-responsive myokine generated by cleavage of fibronectin type III domain-containing protein 5 (FNDC5), and is considered an important candidate molecule linking exercise adaptation, energy metabolism, and immune-inflammatory regulation [50]. Classical studies have shown that skeletal muscle peroxisome proliferator-activated receptor gamma coactivator 1-alpha (PGC-1α) can upregulate FNDC5 expression and promote irisin release, thereby inducing the browning of white adipose tissue, promoting uncoupling protein 1 (UCP1) expression, and contributing to increased energy expenditure and improved glucose homeostasis [50]. Human studies suggest that acute exercise can increase circulating irisin levels, and that irisin may participate in the regulation of skeletal muscle glucose and lipid metabolism through AMP-activated protein kinase (AMPK) activation [51,52]. At present, evidence regarding the effects of irisin on macrophages is mainly derived from animal models, cell experiments, or disease models, and its in vivo causal role under exercise intervention conditions still requires further validation.

Meteorin-like protein (METRNL), also known as Meteorin-like or Meteorin-β, is a secreted protein associated with exercise adaptation and immunometabolic regulation [53,54]. Preclinical studies indicate that exercise can increase METRNL expression in skeletal muscle; however, its macrophage-related effects appear to be mediated mainly through an indirect inter-tissue network rather than through direct macrophage activation. METRNL may enhance eosinophil-associated interleukin-4 (IL-4)/interleukin-13 (IL-13) signaling in adipose tissue, followed by interleukin-4 receptor alpha (IL-4Rα)–signal transducer and activator of transcription 6 (STAT6) activation in macrophages and the reinforcement of repair-associated or alternatively activated macrophage programs [55,56,57,58,59]. Nevertheless, a complete human exercise–skeletal muscle–METRNL–macrophage causal pathway has not yet been established.

Musclin/Ostn, also known as osteocrin, is an exercise-responsive myogenic secretory peptide encoded by the *Ostn* gene and was initially identified as a skeletal muscle-derived secretory factor [60]. Musclin potentiates natriuretic peptide signaling by competing for natriuretic peptide receptor C (NPR-C)-mediated clearance, thereby preserving atrial natriuretic peptide (ANP) availability and enhancing cyclic guanosine monophosphate (cGMP) signaling. Human data show parallel increases in musclin and ANP during exercise, although this association is not causal [61,62]. Preclinical studies further implicate exercise-induced musclin in fibroadipogenic progenitor regulation and muscle homeostasis [63]. In humans, serum musclin increases during moderate-intensity aerobic exercise and early recovery, but muscle-specific release and macrophage effects remain untested [62].

Fibroblast growth factor 21 (FGF21) and β-aminoisobutyric acid (BAIBA) are included here as exercise-responsive metabolic signals rather than as typical protein myokines, because their tissue origins, molecular properties, and modes of action extend beyond classical skeletal muscle-derived signaling. FGF21 has endocrine and paracrine actions and can be produced by multiple tissues, including the liver, adipose tissue, myocardium, and skeletal muscle [64,65]. Acute exercise can induce a transient increase in circulating FGF21 levels, but its source is influenced by exercise mode, energy status, and metabolic background, with hepatic production playing an important role in multiple contexts [66]. Therefore, an exercise-induced increase in circulating FGF21 should not be directly equated with a skeletal muscle-specific myokine effect. BAIBA is an exercise-responsive small-molecule metabolite. Receptor-level studies have linked L-β-aminoisobutyric acid (L-BAIBA) to MAS-related G protein-coupled receptor D (MRGPRD) in selected target cells, whereas its metabolic effects, including white adipose tissue browning and hepatic fatty acid β-oxidation, are mainly associated with peroxisome proliferator-activated receptor alpha (PPARα)-dependent signaling; AMPK-dependent effects have also been reported in metabolic disease models [67,68]. These mechanisms should not be attributed to G protein-coupled receptor 109A (GPR109A), and direct evidence for BAIBA-mediated macrophage regulation remains limited. Overall, compared with irisin and METRNL, FGF21 and BAIBA more strongly reflect systemic energy metabolism, redox regulation, and remodeling of the tissue metabolic microenvironment. Their inclusion in this review is therefore based on their potential immunometabolic relevance, while evidence that they directly mediate skeletal muscle–macrophage communication remains limited.

### 2.4. Matrix Remodeling and Growth/Repair-Related Factors: Indirect Signals Shaping the Repair Niche

The effects of exercise-induced skeletal muscle secretory signals on the macrophage functional spectrum are not limited to direct receptor–signaling pathway interactions. Factors related to matrix remodeling, growth regulation, and angiogenesis may indirectly shape the repair niche required for macrophage functional transitions by altering extracellular matrix architecture, tissue mechanical properties, muscle regenerative capacity, and local oxygen supply.

Myostatin, also known as growth differentiation factor 8 (GDF-8), is a member of the transforming growth factor beta (TGF-β) superfamily and a classical negative regulator of skeletal muscle growth and myofiber hypertrophy [69]. Exercise interventions can modulate Myostatin expression or signaling activity; however, these changes are influenced by exercise modality, training duration, and sampling time point, and therefore should not be simply summarized as a consistent decrease [70]. Myostatin may indirectly alter the macrophage repair niche by regulating satellite-cell activation, muscle regeneration, and fibrosis [71,72].

Decorin and secreted protein acidic and rich in cysteine (SPARC) are extracellular matrix-associated secretory proteins that have received increasing attention as regulatory molecules involved in exercise-induced matrix remodeling responses. Decorin belongs to the small leucine-rich proteoglycan family and is mainly involved in collagen fibril assembly, extracellular matrix organization, and the regulation of TGF-β/Myostatin-related signaling. It may also be influenced by muscle contraction or training status and contribute to muscle hypertrophy, regeneration, and repair microenvironment remodeling [73]. SPARC, also known as secreted protein acidic and rich in cysteine, is a typical matricellular protein with exercise-responsive properties and is mainly involved in extracellular matrix remodeling, cell adhesion, and tissue adaptation [74]. Decorin and SPARC are more likely to indirectly influence macrophage recruitment, inflammation resolution, and tissue remodeling by regulating matrix architecture, collagen deposition, fibrosis, and the local repair environment.

Insulin-like growth factor 1 (IGF-1) and vascular endothelial growth factor A (VEGF-A) are primarily involved in post-exercise skeletal muscle regeneration and angiogenesis. IGF-1 can promote satellite cell activation, myoblast proliferation and differentiation, and protein synthesis, and may support inflammation resolution and muscle regeneration during skeletal muscle injury repair [75,76]. VEGF-A mainly promotes angiogenesis and improves local oxygen supply and nutrient exchange, thereby providing a structural basis for tissue repair and macrophage-mediated tissue remodeling [77]. Overall, IGF-1 and VEGF-A are more likely to indirectly influence macrophage recruitment, inflammation resolution, and repair-supportive functions by optimizing muscle regeneration, vascularization, and tissue remodeling conditions.

### 2.5. Extracellular Vesicles and Non-Coding Ribonucleic Acids: Emerging Carriers of Muscle–Immune Communication

Extracellular vesicles (EVs) and their non-coding ribonucleic acid (RNA) cargo provide an emerging explanatory framework for exercise-induced muscle–immune communication. Exercise can alter the abundance, subpopulation characteristics, and molecular cargo composition of circulating and skeletal muscle-associated EVs, including functional molecules such as microRNAs (miRNAs), proteins, and lipids [10,78,79]. Compared with single myokines, EVs may regulate recipient cell states through coordinated multi-component signaling and participate in inflammatory responses, metabolic adaptation, oxidative stress regulation, and tissue repair [79]. Existing cell-based studies suggest that skeletal muscle myotube-derived EVs can attenuate macrophage inflammatory responses, possibly in association with activation of the immune-responsive gene 1 (IRG1)–itaconate immunometabolic axis [80]. However, research on exercise-induced EVs still faces several challenges, including complex circulating origins, insufficient skeletal muscle-specific markers, limited in vivo evidence for macrophage targeting, and inconsistent standards for EV isolation and characterization. Overall, EVs and their non-coding RNAs provide a new perspective for understanding exercise-induced interactions between muscle and immune cells. Nevertheless, evidence for their direct regulation of the macrophage functional spectrum remains relatively limited, and the relevant mechanisms require further confirmation through skeletal muscle-source tracing, cell-targeting validation, and in vivo functional experiments.

In summary, the evidence supporting the role of exercise-induced skeletal muscle secretory factors in regulating macrophage functional remodeling shows clear hierarchical differences. The evidence hierarchy of representative exercise-induced skeletal muscle secretory factors involved in macrophage functional remodeling is summarized in Table 1. The strength and completeness of the evidence differ substantially among these factors. Human exercise-response evidence is available for IL-6 and irisin, whereas evidence for METRNL is derived mainly from preclinical studies and supports an indirect immunometabolic network rather than a complete human exercise–skeletal muscle–macrophage causal pathway. In contrast, signals such as FGF21, BAIBA, Decorin, Myostatin, IGF-1, VEGF-A, and EVs/miRNAs appear to act more indirectly through systemic metabolic improvement, tissue microenvironment remodeling, or intercellular communication. Therefore, the exercise-induced secretory network should not be viewed as a single driver of macrophage polarization. Rather, it may collectively influence the macrophage functional spectrum through multiple interconnected processes, including immune recruitment, temporal regulation of inflammation, immunometabolic reprogramming, and repair-niche formation.

Taken together, these secretory signals may be conceptualized along an overlapping temporal continuum rather than viewed as isolated molecular categories [19,20,81,82]. During exercise and the first minutes to hours of recovery, chemokines and rapidly responsive cytokines primarily coordinate immune-cell mobilization, recruitment, and the establishment of an early inflammatory microenvironment [23,26,27,28,29,40,41,83,84,85,86,87]. During the subsequent recovery period, broadly spanning hours to days, immunoregulatory signals and pathway crosstalk contribute to phagocytosis, efferocytosis, inflammation resolution, and the progressive transition toward repair-supportive macrophage functions [19,81,82,87,88,89,90]. Although several metabolic factors may also respond acutely, their cumulative macrophage-related effects may become more apparent with repeated exercise over days to weeks, during which AMPK-, nuclear factor erythroid 2-related factor 2 (Nrf2)-, STAT6-, and peroxisome proliferator-activated receptor gamma (PPARγ)-associated programs support immunometabolic remodeling, oxidative adaptation, and the restriction of persistent inflammatory signaling [5,6,88,89,91,92,93]. During later tissue-remodeling phases and with longer-term training adaptation, matrix-, angiogenesis-, and growth-related factors may contribute to extracellular matrix remodeling, vascularization, myofiber regeneration, and stabilization of the regenerative niche [75,76,77,94,95,96,97]. These temporal windows are approximate and substantially overlapping rather than strictly sequential, and their magnitude and duration depend on exercise modality, load, training status, tissue damage, sampling time, tissue type, and disease context.

## 3. Mechanistic Integration of Exercise-Induced Skeletal Muscle Secretory Factors in Regulating the Macrophage Functional Spectrum

The effects of exercise-induced skeletal muscle secretory networks on the macrophage functional spectrum are multilayered and context-dependent. Current evidence suggests that this regulatory process is not directly determined by a single myokine, but instead involves multiple mechanistic layers, including receptor-mediated signal transduction, immunometabolic reprogramming, tissue microenvironment remodeling, and temporal- and tissue-specific regulation. Together, these mechanisms may contribute to macrophage recruitment, inflammatory clearance, inflammation resolution, tissue repair support, and the maintenance of metabolic homeostasis.

### 3.1. Dynamic Spectrum of Macrophage Polarization and Its Significance in Exercise Immunology

In the context of exercise immunology, acute exercise, eccentric contraction, or muscle microdamage can induce transient immune cell mobilization, enabling monocytes and macrophages to participate in damage-signal recognition, clearance of necrotic tissue, and early inflammatory responses [19,20,98]. As recovery progresses, macrophage functions gradually shift toward efferocytosis, inflammation resolution, angiogenesis, matrix remodeling, and support of tissue repair [19,99]. This indicates that exercise-associated immune responses are not simply pro-inflammatory or anti-inflammatory processes, but rather temporally coordinated regulatory events closely related to exercise load, recovery stage, and tissue repair demands.

In this context, the effects of exercise-induced skeletal muscle secretory factors on macrophages are not limited to driving a specific phenotypic transition. Instead, these factors may collectively shape the macrophage functional spectrum by regulating inflammatory clearance, immunometabolic status, and tissue repair responses [19,100]. This perspective helps explain the link between transient inflammatory responses after acute exercise and long-term anti-inflammatory adaptation, and may also provide mechanistic guidance for exercise-load management and the determination of optimal recovery windows. Therefore, when evaluating macrophage functional states, phenotypic markers such as inducible nitric oxide synthase (iNOS), cluster of differentiation 86 (CD86), arginase 1 (Arg1), and cluster of differentiation 206 (CD206) should be complemented by functional indicators, including phagocytic capacity, efferocytosis, inflammatory cytokine profiles, metabolic status, and tissue repair outcomes.

### 3.2. Receptor–Signaling Axes: Mechanistic Convergence Points for Distinct Secretory Signals

Exercise-induced skeletal muscle secretory signals may influence the macrophage functional spectrum through multiple pathways, including the gp130/Janus kinase (JAK)/signal transducer and activator of transcription (STAT), STAT6/PPARγ, AMPK/Nrf2/NF-κB, TGF-β/Smad, and chemokine receptor axes. These pathways do not act in isolation, nor do they correspond to individual secretory factors in a simple one-to-one manner. Instead, they form interconnected regulatory networks that operate across different exercise loads, recovery phases, and tissue niches.

#### 3.2.1. gp130/JAK/STAT Axis: Temporal Regulation of Inflammation Initiation and the Transition Toward Repair

**Figure 1:**
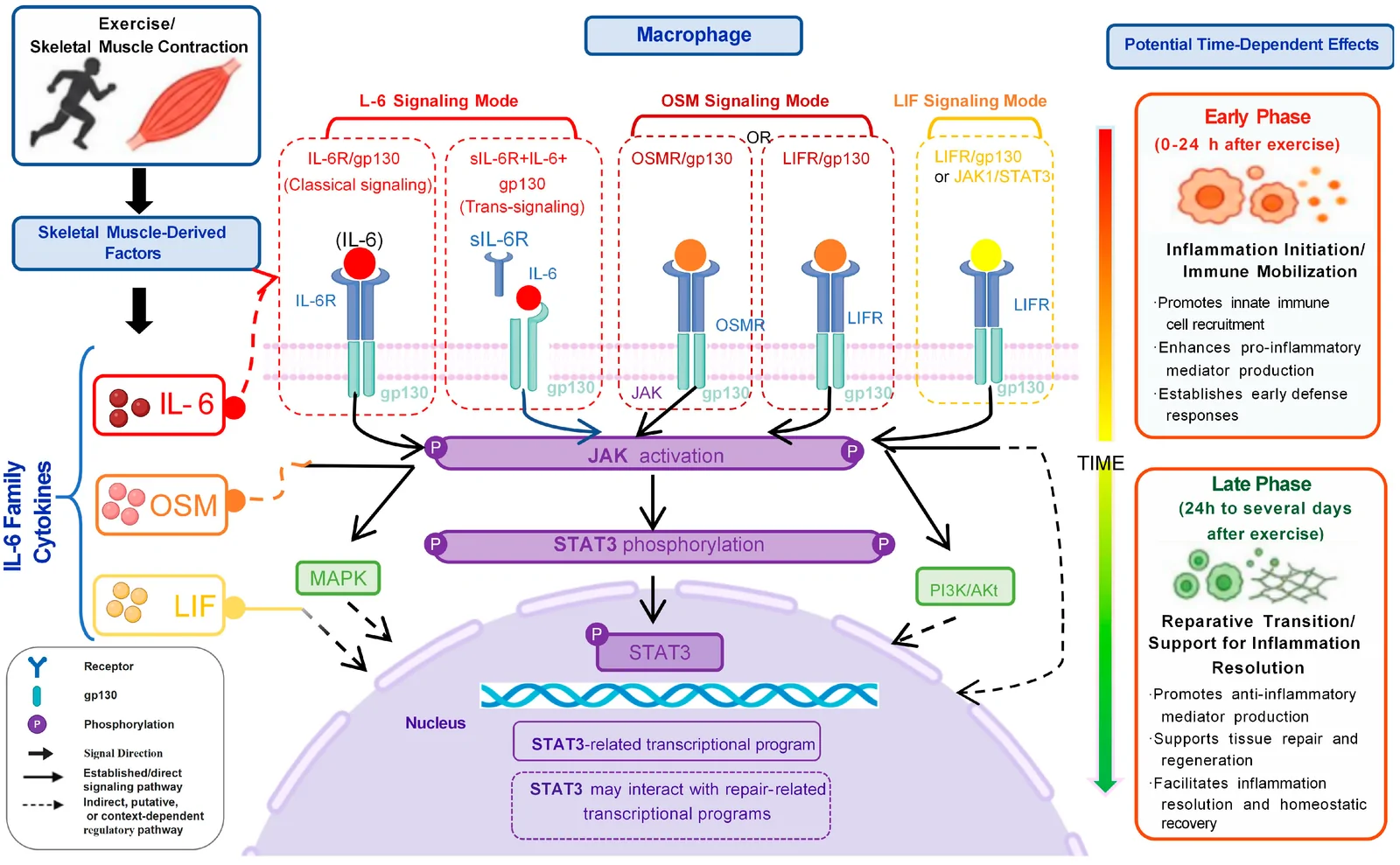
The gp130/JAK/STAT3 axis in exercise-induced macrophage regulation. Created in BioRender. Li, Z. (2026) https://BioRender.com/zdufvoz (accessed on 8 August 2026). Abbreviations: IL-6, interleukin-6; OSM, oncostatin M; LIF, leukemia inhibitory factor; IL-6R, interleukin-6 receptor; sIL-6R, soluble interleukin-6 receptor; gp130, glycoprotein 130; OSMR, oncostatin M receptor; LIFR, leukemia inhibitory factor receptor; JAK, Janus kinase; STAT3, signal transducer and activator of transcription 3; MAPK, mitogen-activated protein kinase; PI3K, phosphoinositide 3-kinase; Akt, protein kinase B.

The gp130/JAK/STAT axis is an important signaling system linking exercise-induced cytokine responses to the regulation of macrophage function. IL-6, OSM, and leukemia inhibitory factor (LIF) all belong to the IL-6 cytokine family and can activate the JAK/STAT pathway through gp130-associated receptor complexes, with further crosstalk with downstream mitogen-activated protein kinase (MAPK) and phosphoinositide 3-kinase/protein kinase B (PI3K/Akt) signaling [101,102,103] (Figure 1). The macrophage effects of IL-6 are stage- and context-dependent. During early inflammation, IL-6 acts within a TNF-α-rich milieu associated with immune-cell recruitment and inflammatory clearance. As pro-inflammatory signals decline, IL-6–signal transducer and activator of transcription 3 (STAT3) signaling may support resolution, homeostasis, and repair-associated macrophage programs [104]. IL-6 alone, however, is insufficient to induce a stable M2 phenotype. OSM primarily supports local recruitment and repair-niche remodeling. OSM mainly acts through oncostatin M receptor (OSMR)/gp130 or leukemia inhibitory factor receptor (LIFR)/gp130 and appears to contribute primarily to local immune-cell recruitment and repair-niche remodeling [105], whereas LIF activates LIFR/gp130–Janus kinase 1 (JAK1)/STAT3 signaling and may support repair-associated transcriptional programs [103]. STAT3 signaling may interact with repair-associated transcriptional programs; however, whether it constitutes a direct mechanism underlying exercise-induced macrophage functional transition toward repair remains to be further validated [106,107]. Therefore, although these cytokines converge on the gp130/JAK/STAT3 axis, their functional effects differ according to receptor usage, temporal stage, and tissue context.

#### 3.2.2. STAT6/PPARγ Axis: Alternative Activation and Anti-Inflammatory Reparative Programs

The STAT6/PPARγ axis is an important regulatory pathway involved in macrophage alternative activation, fatty acid oxidation, and repair-supportive functions. After IL-4 and IL-13 bind to IL-4Rα on the macrophage surface, STAT6 can be activated, leading to the upregulation of repair-associated genes such as *Arg1* and *Mrc1* (encoding arginase 1 and CD206, respectively). Meanwhile, STAT6 can cooperate with metabolic regulators such as PPARγ to promote fatty acid oxidation, mitochondrial oxidative phosphorylation, and anti-inflammatory reparative programs [49,57,58,59].

**Figure 2:**
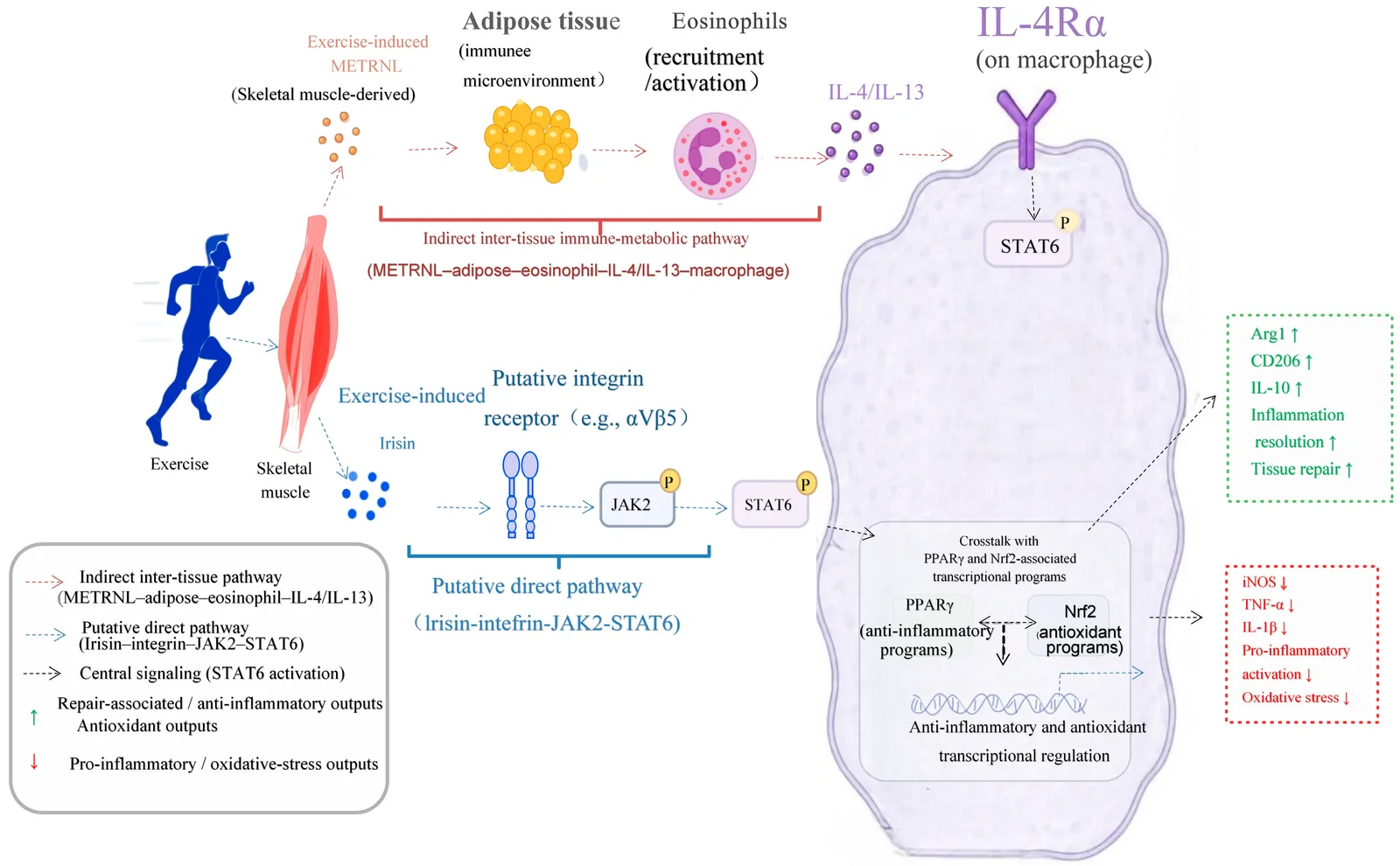
The STAT6/PPARγ axis linking METRNL- and irisin-associated signals to reparative macrophage programs. Abbreviations: METRNL, meteorin-like protein; IL-4, interleukin-4; IL-13, interleukin-13; IL-4Rα, interleukin-4 receptor alpha; JAK2, Janus kinase 2; STAT6, signal transducer and activator of transcription 6; PPARγ, peroxisome proliferator-activated receptor gamma; Nrf2, nuclear factor erythroid 2-related factor 2; Arg1, arginase 1; CD206, cluster of differentiation 206; IL-10, interleukin-10; iNOS, inducible nitric oxide synthase; TNF-α, tumor necrosis factor alpha; IL-1β, interleukin-1 beta.

Among exercise-induced skeletal muscle secretory factors, METRNL and Irisin are closely linked to this axis (Figure 2). Preclinical evidence suggests that METRNL may indirectly promote macrophage STAT6 activation through a multilevel immunometabolic network involving skeletal muscle, adipose tissue, eosinophils, IL-4/IL-13, and macrophages [55,108,109]. Irisin may influence Janus kinase 2 (JAK2)/STAT6 through integrin-related mechanisms and further engage PPARγ- and Nrf2-associated anti-inflammatory and antioxidant transcriptional programs. These effects are reflected by the upregulation of repair-associated markers such as Arg1, CD206, and interleukin-10 (IL-10), together with reduced pro-inflammatory indicators such as iNOS, TNF-α, and IL-1β [110,111]. This axis suggests that exercise-induced secretory factors may affect macrophage function not only through direct receptor-mediated actions, but also indirectly through inter-tissue immune cell interactions.

#### 3.2.3. AMPK/Nrf2/NF-κB Axis: Redox Homeostasis and Pro-Inflammatory Transcriptional Regulation

**Figure 3:**
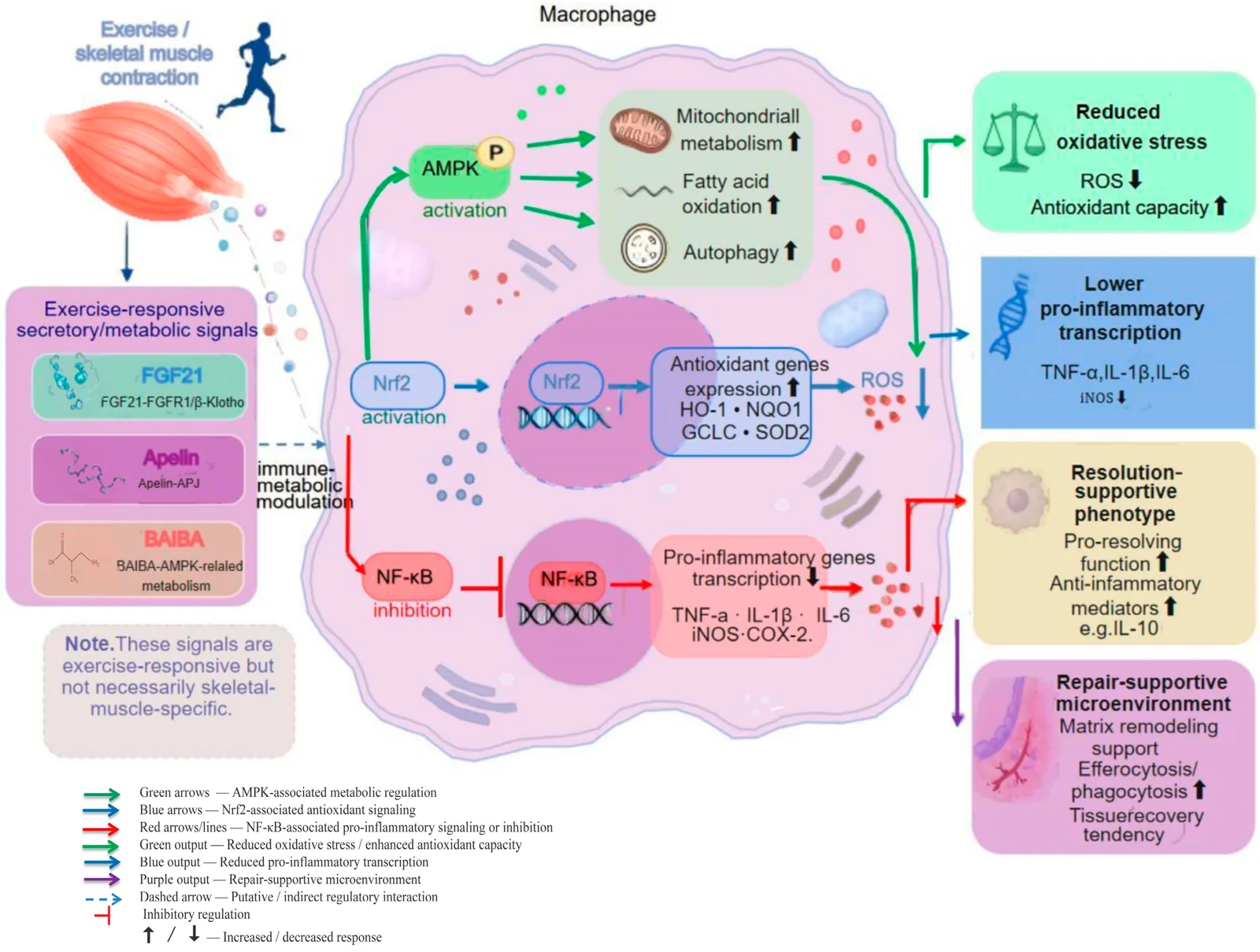
The AMPK/Nrf2/NF-κB axis in macrophage immunometabolic and redox regulation. Abbreviations: AMPK, AMP-activated protein kinase; Nrf2, nuclear factor erythroid 2-related factor 2; NF-κB, nuclear factor kappa B; FGF21, fibroblast growth factor 21; FGFR1, fibroblast growth factor receptor 1; APJ, apelin receptor; BAIBA, β-aminoisobutyric acid; ROS, reactive oxygen species; HO-1, heme oxygenase-1; NQO1, NAD(P)H quinone oxidoreductase 1; TNF-α, tumor necrosis factor alpha; IL-1β, interleukin-1 beta; IL-6, interleukin-6; iNOS, inducible nitric oxide synthase; COX-2, cyclooxygenase-2; IL-10, interleukin-10.

The AMPK/Nrf2/NF-κB axis is a key pathway linking macrophage energy metabolism, redox homeostasis, and inflammatory transcriptional responses. AMPK participates in the regulation of mitochondrial metabolism, fatty acid oxidation, and autophagy [112]. Nrf2 reduces reactive oxygen species (ROS) accumulation by inducing antioxidant defenses, including heme oxygenase-1 (HO-1) and NAD(P)H quinone oxidoreductase 1 (NQO1) [113,114]. NF-κB regulates the expression of pro-inflammatory mediators, including TNF-α, IL-1β, IL-6, and iNOS [112,113,114,115,116]. Together, these pathways determine macrophage functional transitions among pro-inflammatory responses, oxidative stress control, and repair support (Figure 3).

Among exercise-induced secretory signals, factors such as FGF21, Apelin, and BAIBA are associated with energy metabolism, lipid oxidation, and inflammatory regulation [66,68,117,118,119,120]. FGF21 can participate in metabolic and inflammatory regulation through the fibroblast growth factor receptor 1 (FGFR1)/β-Klotho receptor system [121] and has been shown to attenuate macrophage-mediated inflammation by activating Nrf2-dependent antioxidant responses and suppressing NF-κB signaling in lipopolysaccharide (LPS)-stimulated macrophage models [117]. However, because exercise-induced increases in circulating FGF21 may originate substantially from the liver rather than skeletal muscle under certain conditions [66], elevated circulating FGF21 after exercise should not be directly interpreted as a skeletal muscle-specific myokine effect. Therefore, this axis is better understood as a regulatory pathway through which exercise-induced secretory signals mediate immunometabolic reprogramming. Its core role is to reduce pro-inflammatory transcriptional pressure, improve redox status, and provide a metabolic basis for inflammation resolution and tissue repair.

#### 3.2.4. TGF-β/Smad and Matrix Remodeling Axis: Indirect Regulation of the Repair Microenvironment

**Figure 4:**
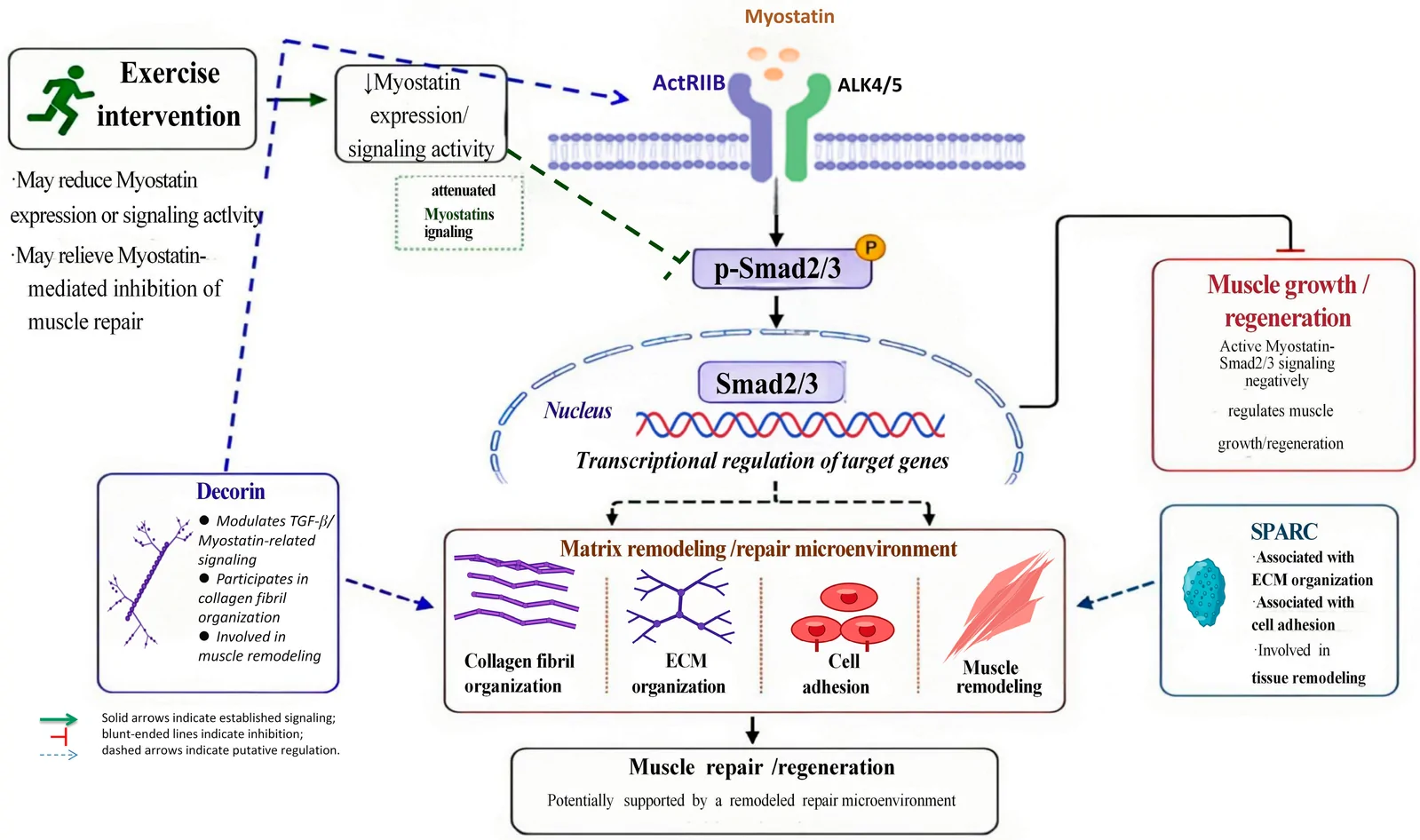
The TGF-β/Smad and matrix remodeling axis in repair-niche regulation. Abbreviations: ActRIIB, activin receptor type IIB; ALK4/5, activin receptor-like kinase 4/5; p-Smad2/3, phosphorylated SMAD family member 2/3; Smad2/3, SMAD family member 2/3; TGF-β, transforming growth factor beta; ECM, extracellular matrix; SPARC, secreted protein acidic and rich in cysteine.

The TGF-β/Smad and matrix remodeling axis mainly influences the local niche in which macrophage functional transitions occur by regulating extracellular matrix deposition, the degree of fibrosis, tissue mechanical properties, and muscle regenerative capacity [122] (Figure 4). Unlike signaling axes that directly regulate macrophage inflammatory transcription or metabolic programs, this axis emphasizes the constraints imposed by the structural environment of the injured region on immune repair processes. Myostatin, a member of the TGF-β superfamily, negatively regulates muscle growth and regeneration through activin receptor type IIB (ActRIIB)/activin receptor-like kinase 4/5 (ALK4/5)–SMAD family member 2/3 (Smad2/3) signaling [123]. Multiple exercise intervention studies suggest that Myostatin expression or signaling activity may decrease, which may help attenuate its inhibitory effects on muscle repair [124,125,126]. Decorin can modulate TGF-β/Myostatin-related signaling and participate in collagen fibril organization and muscle remodeling [127]. SPARC is closely associated with extracellular matrix organization, cell adhesion, and tissue remodeling [128]. The influence of this axis on macrophages is mainly reflected in niche regulation. By modifying matrix architecture, fibrosis levels, and local mechanical conditions, it can affect macrophage migration, inflammation resolution, and repair-supportive functions.

#### 3.2.5. Chemokine Receptor Axis: Immune Cell Recruitment and Establishment of the Pre-Polarization Microenvironment

**Figure 5:**
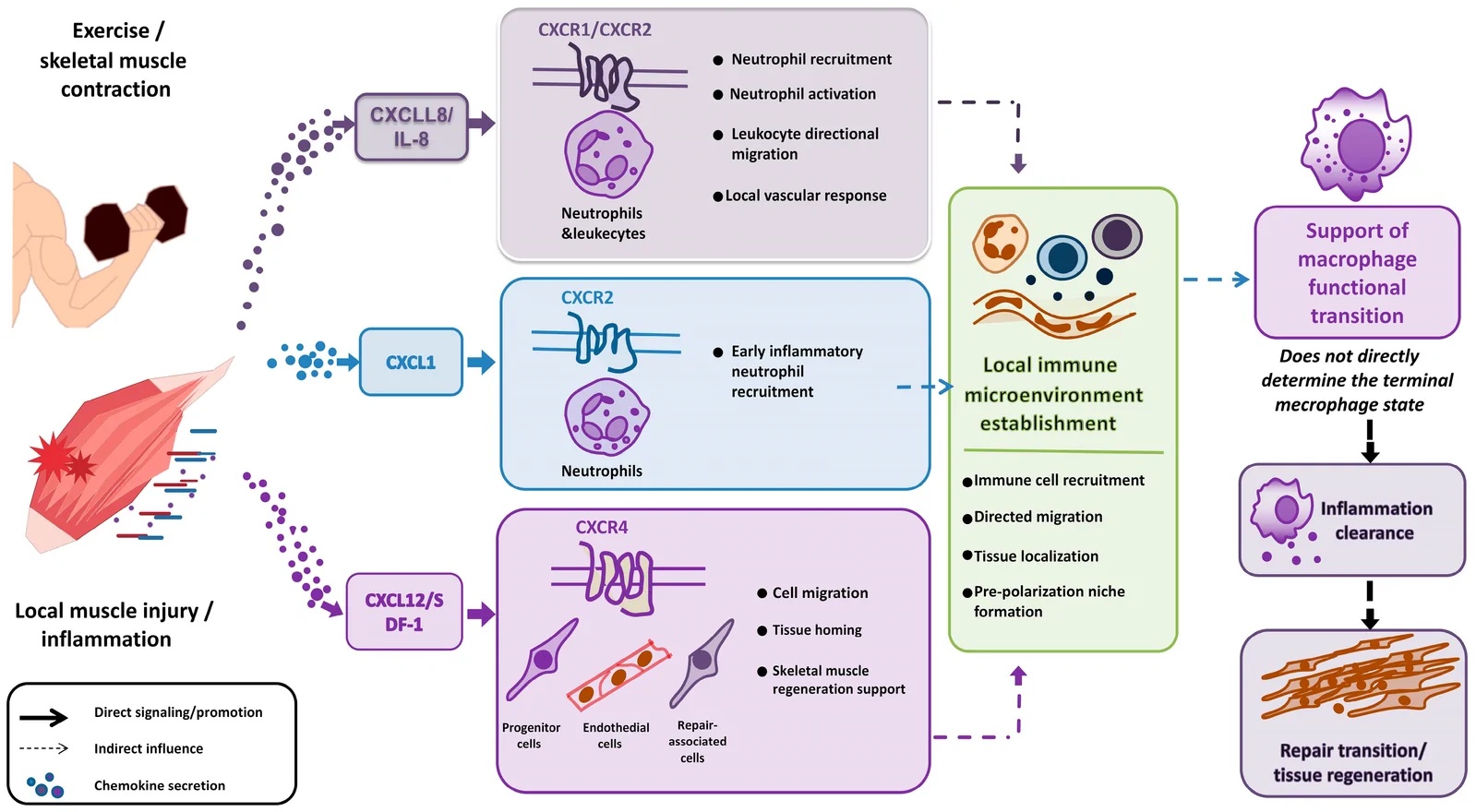
The chemokine receptor axis in immune cell recruitment and pre-polarization niche formation. Abbreviations: CXCL8, C-X-C motif chemokine ligand 8; IL-8, interleukin-8; CXCR1, C-X-C motif chemokine receptor 1; CXCR2, C-X-C motif chemokine receptor 2; CXCL1, C-X-C motif chemokine ligand 1; CXCL12, C-X-C motif chemokine ligand 12; SDF-1, stromal cell-derived factor 1; CXCR4, C-X-C motif chemokine receptor 4.

The chemokine receptor axis mainly regulates immune cell recruitment, migration, and tissue positioning, and represents an important mechanism for establishing the local immune microenvironment after exercise. CXCL8/IL-8 mainly mediates neutrophil recruitment and activation through CXCR1/CXCR2 and participates in leukocyte directional migration during injury and inflammatory responses [129]. Exercise can induce IL-8 expression and CXCR2-related responses in human skeletal muscle, suggesting that this pathway may participate in post-exercise local vascular responses and immune cell recruitment [29]. CXCL1 mainly contributes to early neutrophil recruitment through CXCR2 during inflammation [83,84]. CXCL12/SDF-1 regulates the migration of progenitor cells, endothelial cells, and repair-associated cells through CXCR4 and participates in skeletal muscle regeneration [31]. The chemokine axis does not directly determine the terminal functional states of macrophages. Instead, by regulating immune cell recruitment, directional migration, and tissue positioning, it shapes the local immune microenvironment required for macrophage functional transitions, thereby providing the cellular basis for inflammatory clearance and the subsequent transition toward repair (Figure 5) [85].

#### 3.2.6. Crosstalk and Temporal Coordination Among Signaling Pathways

These signaling pathways do not operate as isolated or strictly sequential modules but form an interconnected regulatory network through shared transcriptional, metabolic, and redox-sensitive nodes. In the context of tissue stress or muscle damage, toll-like receptor 4 (TLR4) can activate myeloid differentiation primary response 88 (MyD88)- and TIR-domain-containing adapter-inducing interferon-β (TRIF)-dependent signaling. Interleukin-1 receptor-associated kinase 4 (IRAK4)-dependent signaling subsequently promotes tumor necrosis factor receptor-associated factor 6 (TRAF6) activation, whereas downstream activation of the IκB kinase (IKK)–inhibitor of κB (IκB)–NF-κB cascade induces the expression of inflammatory cytokines and chemokines [130,131,132]. IL-6 released within the injured-muscle microenvironment can engage gp130/JAK/STAT3 signaling and contribute to macrophage chemokine expression and recruitment [133]. Functional interactions between STAT3 and NF-κB may further reshape the magnitude and duration of inflammatory transcription [134]. Suppressor of cytokine signaling 3 (SOCS3)-mediated negative feedback limits excessive or prolonged IL-6-induced JAK/STAT activation [135].

As recovery progresses, STAT6–PPARγ/peroxisome proliferator-activated receptor gamma coactivator 1-beta (PGC-1β)-associated programs promote fatty acid oxidation, mitochondrial biogenesis, and repair-associated transcription, whereas AMP-activated protein kinase alpha 1 (AMPKα1) supports phagocytosis- and efferocytosis-associated macrophage transitions during skeletal muscle regeneration. Nrf2-related programs further contribute to antioxidant defense and the restriction of persistent pro-inflammatory transcription [88,136,137]. In parallel, TGF-β/Smad signaling and the interactions among transforming growth factor beta 1 (TGF-β1), Myostatin, and Decorin regulate extracellular matrix deposition and fibrosis, while transforming growth factor beta 1 (TGF-β1) supports vascularization and muscle regeneration. Chemokine receptor signaling, particularly the C-C motif chemokine ligand 2 (CCL2)/C-C motif chemokine receptor 2 (CCR2) axis, contributes to monocyte/macrophage recruitment and positioning within injured muscle [94,95]. Together, these processes may facilitate the transition from inflammatory clearance toward resolution and tissue repair. However, transcriptomic and spatiotemporal studies indicate that inflammatory, resolution-related, vascular, and matrix-remodeling programs substantially overlap rather than forming strictly discrete phases, and their magnitude and timing depend on the extent of tissue damage and the recovery interval [81,82,87].

### 3.3. Immunometabolic Reprogramming: The Metabolic Basis of Macrophage Functional Transitions

Macrophage functional transitions are reflected not only by changes in phenotypic markers, but also by the remodeling of metabolic programs, including glycolysis, mitochondrial oxidative phosphorylation, fatty acid oxidation, and redox homeostasis.

Current studies indicate that efferocytosis represents an important functional link between macrophage metabolic reprogramming and inflammation resolution. This process may promote anti-inflammatory responses and tissue repair through fatty acid oxidation, the electron transport chain, and nicotinamide adenine dinucleotide (NAD^+^)-related metabolic signaling [89]. Studies of skeletal muscle injury also suggest that inflammatory monocytes recruited during the early phase can transition toward repair-supportive states within the local microenvironment and participate in myofiber regeneration [99].

Recent evidence suggests that exercise-induced Musclin release may mediate macrophage metabolic reprogramming through an a formyl peptide receptor 2 (FPR2)-related mechanism, enhance efferocytosis, reduce inflammatory burden, and improve the infection-associated inflammatory microenvironment [91]. By contrast, Irisin appears to mainly regulate anti-inflammatory and antioxidant signaling through the JAK2/STAT6–PPARγ/Nrf2 axis, although sufficient evidence from oxygen consumption rate (OCR)/extracellular acidification rate (ECAR) assays and metabolic blockade experiments remains lacking. Overall, immunometabolic reprogramming may represent an important mechanism by which skeletal muscle secretory factors regulate macrophage functional transitions. However, this mechanism requires further validation through receptor blockade, metabolic flux analysis, and functional outcome assessment.

### 3.4. Tissue Microenvironment Remodeling: Niche Mechanisms Indirectly Regulating Macrophage Functional States

Macrophages are highly sensitive to the local microenvironment. Their functional states are regulated not only by soluble factors, but also by physical cues such as tissue stiffness, matrix architecture, and mechanical stimulation [138]. Studies have shown that Piezo1 can function as a mechanosensitive channel through which macrophages sense matrix stiffness. Matrix stiffening can enhance pro-inflammatory programs and suppress repair-associated functional states through Piezo1–Yes-associated protein (YAP) mechanotransduction [139]. Therefore, changes in the extracellular matrix and mechanical environment of skeletal muscle after exercise or injury may indirectly influence inflammatory clearance and repair-supportive functions by reshaping the niche in which macrophages operate. Skeletal muscle secretory factors may further shape the local conditions required for macrophage functional transitions by regulating matrix remodeling, fibrosis, and angiogenesis. Decorin can be released by contracting myotubes and is increased after acute resistance exercise, participating in skeletal muscle hypertrophy and remodeling [73]. Myostatin can promote skeletal muscle fibroblast proliferation and extracellular matrix protein production, thereby contributing to skeletal muscle fibrosis [140]. Abnormal collagen deposition and fibrosis may disrupt the normal regenerative microenvironment and are associated with imbalanced macrophage polarization and impaired repair [96]. Meanwhile, VEGF can improve tissue regeneration and reduce fibrosis after acute skeletal muscle injury [97]. VEGF/vascular endothelial growth factor receptor 2 (VEGFR-2) signaling is spatially associated with macrophage infiltration in regenerating skeletal muscle regions after ischemia [141], suggesting that angiogenesis, improved oxygen supply, and immune cell infiltration may jointly contribute to reconstruction of the repair microenvironment. Fibroadipogenic progenitors, myogenic extracellular vesicles, and efferocytosis may also serve as complementary mechanisms through which the skeletal muscle microenvironment regulates macrophage function.

### 3.5. Temporal Windows and Tissue Specificity: Key Conditions Determining the Direction of Macrophage Functional Remodeling

Macrophage functional remodeling in the context of exercise is not a fixed pro-inflammatory or anti-inflammatory response. Instead, it is jointly influenced by exercise modality, degree of injury, recovery phase, and tissue microenvironment, showing clear temporal-window dependence and tissue specificity [19,90]. Studies of skeletal muscle injury and regeneration have shown that myeloid cells enter injured regions in a sequential manner after acute injury. During the early phase, they mainly participate in inflammatory responses, necrotic fiber clearance, and phagocytosis of cellular debris; subsequently, they gradually shift toward inflammation resolution, satellite cell regulation, and tissue repair support [19,90].

Specifically, unaccustomed exercise, especially exercise involving substantial eccentric contractions, is more likely to induce exercise-induced muscle damage, characterized by reduced muscle strength, pain, swelling, and increased circulating markers of muscle damage [20,142]. After this type of exercise, damaged myofibers and the local matrix environment can promote the entry of neutrophils, monocytes, and macrophages into exercised muscle regions, where they participate in necrotic tissue clearance and local inflammatory regulation [86]. Human eccentric exercise studies indicate that leukocyte accumulation in muscle follows a defined time course, and sampling time can markedly influence the observed inflammatory cell response.

From a tissue-specific perspective, eccentric contractions and resistance loading primarily act on local skeletal muscle, and the associated macrophage responses are often related to mechanical tension, myofiber microdamage, and regenerative repair [20,142]. By contrast, endurance training more commonly reflects long-term remodeling of the immune microenvironment in metabolic tissues. Walton et al. found that 12 weeks of endurance cycling training increased repair-associated macrophages in the human vastus lateralis muscle, and these changes were associated with alterations in myofiber cross-sectional area and satellite cell number [92]. Kawanishi et al. reported that 16 weeks of treadmill training reduced macrophage infiltration in adipose tissue from obese mice and promoted a shift from an M1-like to an M2-like phenotype [93]. In addition, exercise intensity, duration, training familiarity, and recovery interval can influence the initial degree of muscle damage and the duration of inflammation. Therefore, when analyzing the effects of exercise-induced skeletal muscle secretory networks on macrophage function, exercise modality, load intensity, training duration, sampling time, tissue source, and disease background should be clearly defined.

In summary, exercise-induced skeletal muscle factors regulate macrophage function through four interconnected processes: inflammatory signaling and immune-cell recruitment, immunometabolic reprogramming, repair-niche remodeling, and context-dependent temporal control. Their dominant roles vary by disease context: inflammatory clearance and repair in muscle injury, control of low-grade inflammation in metabolic disease, preservation of regenerative capacity in aging, and context-dependent immune modulation in cancer.

**Figure 6:**
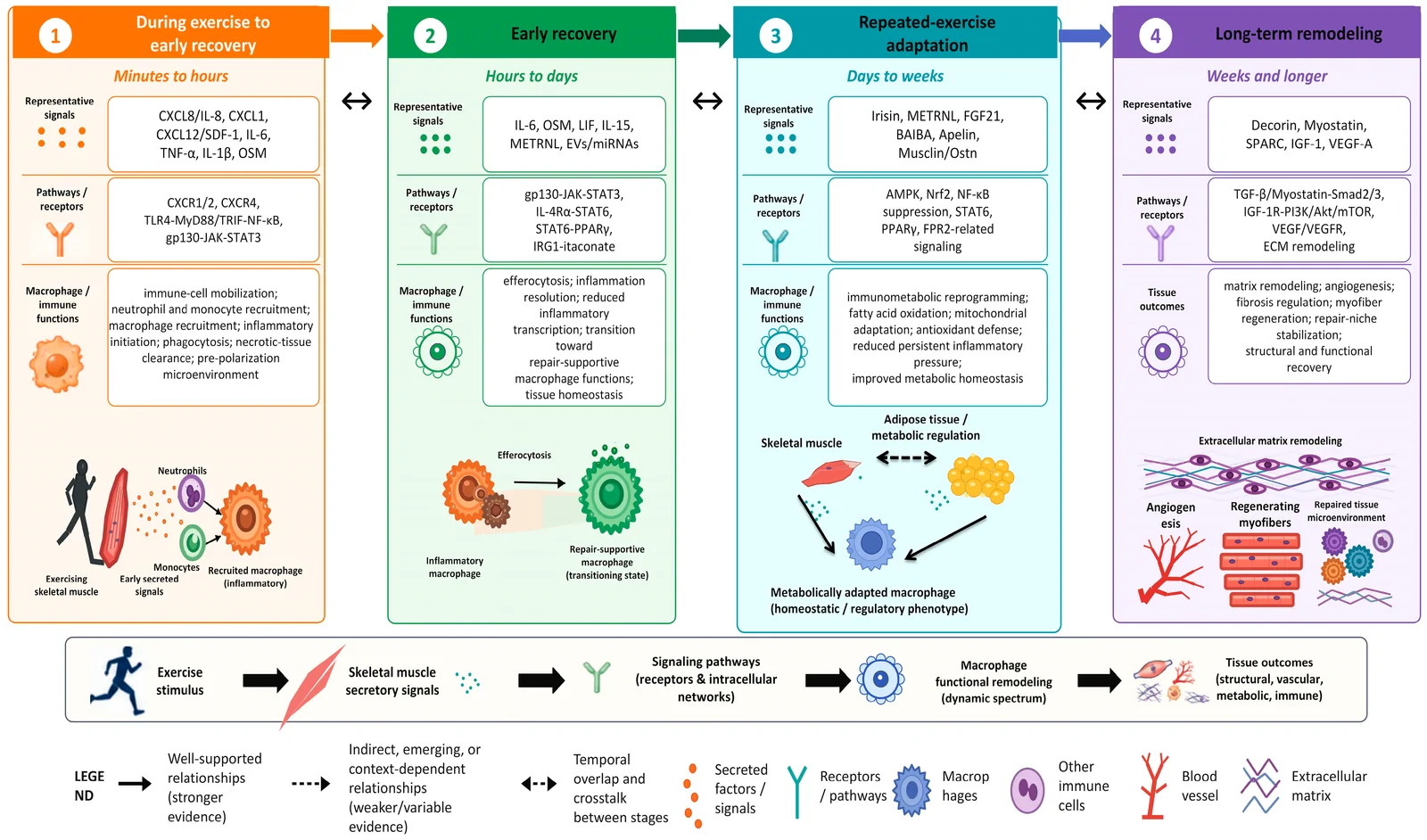
Temporal integration of exercise-induced skeletal muscle secretory signals in macrophage functional remodeling. Abbreviations: CXCL8, C-X-C motif chemokine ligand 8; IL-8, interleukin-8; CXCL1, C-X-C motif chemokine ligand 1; CXCL12, C-X-C motif chemokine ligand 12; SDF-1, stromal cell-derived factor 1; IL-6, interleukin-6; TNF-α, tumor necrosis factor alpha; IL-1β, interleukin-1 beta; OSM, oncostatin M; CXCR1/2, C-X-C motif chemokine receptors 1/2; CXCR4, C-X-C motif chemokine receptor 4; TLR4, toll-like receptor 4; MyD88, myeloid differentiation primary response 88; TRIF, TIR-domain-containing adapter-inducing interferon-β; NF-κB, nuclear factor kappa B; gp130, glycoprotein 130; JAK, Janus kinase; STAT3, signal transducer and activator of transcription 3; LIF, leukemia inhibitory factor; IL-15, interleukin-15; METRNL, meteorin-like protein; EVs, extracellular vesicles; miRNAs, microRNAs; IL-4Rα, interleukin-4 receptor alpha; STAT6, signal transducer and activator of transcription 6; PPARγ, peroxisome proliferator-activated receptor gamma; IRG1, immune-responsive gene 1; FGF21, fibroblast growth factor 21; BAIBA, β-aminoisobutyric acid; AMPK, AMP-activated protein kinase; Nrf2, nuclear factor erythroid 2-related factor 2; FPR2, formyl peptide receptor 2; SPARC, secreted protein acidic and rich in cysteine; IGF-1, insulin-like growth factor 1; VEGF-A, vascular endothelial growth factor A; TGF-β, transforming growth factor beta; Smad2/3, SMAD family member 2/3; PI3K, phosphoinositide 3-kinase; Akt, protein kinase B; mTOR, mechanistic target of rapamycin; VEGF, vascular endothelial growth factor; VEGFR, vascular endothelial growth factor receptor; ECM, extracellular matrix.

From a temporal perspective, these pathways can be interpreted as an overlapping and coordinated regulatory cascade. Chemokine receptor signaling and TLR4/NF-κB-related inflammatory programs are particularly relevant to early immune-cell recruitment and inflammatory clearance. During early recovery, gp130/JAK/STAT3 and STAT6/PPARγ signaling contribute to the regulation of inflammation resolution, efferocytosis, and repair-supportive macrophage functions. With repeated exercise, AMPK/Nrf2/NF-κB-related signaling increasingly supports metabolic and redox adaptation, whereas TGF-β/Smad-, IGF-1/PI3K/Akt-, and VEGF-related pathways contribute to longer-term extracellular matrix remodeling, vascularization, and repair-niche stabilization. Importantly, these signaling modules interact extensively and should not be interpreted as strictly separated or sequential phases (Figure 6).

## 4. Clinical Translational Implications of Exercise-Induced Skeletal Muscle Secretory Factors in Regulating Macrophage Functional Remodeling

From a clinical translational perspective, the significance of exercise-induced skeletal muscle secretory networks in shaping the macrophage functional spectrum should be interpreted within specific disease contexts. The major macrophage-related challenges, relevant secretory signals, mechanisms of action, exercise prescription variables, and evaluation indicators differ across clinical scenarios. Therefore, “anti-inflammatory” or “pro-reparative” effects should not be used as a uniform explanatory framework.

### 4.1. Skeletal Muscle Injury and Postoperative Rehabilitation: The Temporal Window of Inflammatory Clearance–Repair Transition and Load Control

In the context of skeletal muscle injury and postoperative rehabilitation, the key issue in macrophage functional remodeling is not simply the suppression of inflammation, but the orderly coordination of inflammatory clearance and tissue repair within an appropriate temporal window. During the early stage of injury, inflammatory monocytes/macrophages mainly participate in the phagocytic clearance of necrotic myofibers, cellular debris, and damage-associated signals. As repair progresses, these cells gradually shift toward anti-inflammatory and repair-supportive states, thereby contributing to satellite cell regulation, myofiber regeneration, angiogenesis, matrix remodeling, and restoration of tissue homeostasis [99,143,144]. In an eccentric contraction-induced muscle injury model in older adults, spatial transcriptomic and single-cell transcriptomic analyses revealed dynamic changes in cell populations associated with immune responses, matrix remodeling, and myofiber regeneration across different recovery stages after injury. Animal studies also suggest that appropriate mechanical loading can influence inflammatory responses, macrophage phenotype transitions, and the progression of fibrosis in injured muscle [145,146]. These findings indicate that exercise interventions during the early stage of injury should avoid premature or excessive suppression of necessary inflammatory responses. Instead, prescription design should focus on load control, reduction in secondary injury, and safe restoration of physical activity.

Exercise-induced changes in gp130-related cytokines, such as IL-6, OSM, and LIF, together with alterations in IGF-1, VEGF-A, Decorin, and Myostatin signaling, may indirectly shape the local niche for macrophage functional transitions by regulating immune cell recruitment, muscle regeneration, vascularization, and the extracellular matrix environment. Therefore, the evaluation of injury rehabilitation should integrate multidimensional outcomes, including inflammatory clearance, satellite cell activation, myofiber regeneration, collagen alignment, pain relief, range of motion, and recovery of muscle strength, rather than relying solely on phenotypic markers such as CD86, iNOS, CD206, or Arg1.

### 4.2. Metabolic Diseases: Control of Chronic Low-Grade Inflammation and Stratification of Exercise Prescriptions

Obesity, type 2 diabetes, and metabolic dysfunction-associated fatty liver disease are often accompanied by chronic low-grade inflammation, insulin resistance, and abnormal macrophage infiltration in adipose tissue or the liver [147]. Animal studies have shown that regular exercise can attenuate high-fat diet-induced adipose tissue inflammation, reduce pro-inflammatory macrophage infiltration, and promote a shift in adipose tissue macrophages from an M1-like toward an M2-like phenotype. Exercise training can also alleviate hepatic inflammation, fibrosis, and macrophage infiltration in diet-induced obese mice [93,148]. From the perspective of exercise prescription design, aerobic exercise mainly contributes to improving cardiorespiratory fitness, glycemic control, and lipid metabolism, whereas resistance training may help increase skeletal muscle mass, enhance muscle strength, and improve insulin sensitivity [149]. Exercise-responsive factors such as Irisin, METRNL, FGF21, BAIBA, and Apelin may serve as candidate molecular markers of immunometabolic adaptation. However, they should not currently be used alone to determine exercise efficacy. A more appropriate approach is to evaluate these factors together with glucose metabolism, lipid profiles, insulin sensitivity, body composition, inflammatory mediators, cardiorespiratory fitness, and muscle strength, thereby providing an integrated assessment of the effects of exercise prescriptions on metabolic homeostasis and chronic inflammatory status [150].

### 4.3. Aging and Sarcopenia: Inflammaging, Degeneration of the Regenerative Niche, and Maintenance of Functional Reserve

Aging and sarcopenia are not characterized solely by the loss of skeletal muscle mass, but also involve chronic low-grade inflammation, impaired muscle regenerative capacity, and dysregulation of the immune microenvironment. Studies have shown that aged skeletal muscle exhibits abnormalities in macrophage recruitment, macrophage abundance, and phenotypic transitions during recovery from disuse or injury, which may affect satellite cell activation, inflammation resolution, matrix remodeling, and myofiber regeneration [143,151]. Recent studies on myokine networks suggest that exercise-induced secretory signals may exert cross-system regulatory effects across the musculoskeletal, metabolic, cardiovascular, nervous, and immune systems by modulating core aging-related processes, including chronic inflammation, metabolic imbalance, and tissue homeostatic dysfunction [152]. For example, exercise-induced brain-derived neurotrophic factor (BDNF) may participate in skeletal muscle metabolic recovery and tissue remodeling during the post-exercise recovery phase through peroxisome proliferator-activated receptor delta (PPARδ)-dependent lipid metabolic reprogramming, providing specific evidence for the relationship among exercise-responsive factors, metabolic adaptation, and maintenance of muscle function [153]. Changes in IL-15, IGF-1, Irisin, Decorin, and Myostatin signaling are associated with exercise adaptation, muscle anabolism, and maintenance of muscle mass. However, current evidence does not demonstrate that changes in a single circulating myokine are sufficient to represent improvement in sarcopenia [154]. In clinical translation, functional indicators proposed by the Asian Working Group for Sarcopenia (AWGS) 2019, including muscle mass, handgrip strength, gait speed, the five-time chair stand test, the Short Physical Performance Battery, and the Timed Up and Go test, should remain the core evaluation measures [155]. Candidate secretory factors may be incorporated as supplementary indicators for mechanistic interpretation and efficacy monitoring, thereby facilitating the transition of exercise prescriptions from experience-based arrangements toward function-stratified interventions.

### 4.4. Cancer Rehabilitation: Potential for Immune Microenvironment Modulation and Safety Boundaries

Cancer rehabilitation should be clearly distinguished from skeletal muscle injury repair. In tissue repair, repair-associated macrophages contribute to inflammation resolution and tissue reconstruction. In contrast, within the tumor microenvironment, tumor-associated macrophages (TAMs), particularly immunosuppressive or M2-like TAMs, are often associated with aberrant angiogenesis, matrix remodeling, invasion and metastasis, suppression of antitumor immunity, and treatment resistance [156]. Therefore, the effects of exercise-induced skeletal muscle secretory factors should not be interpreted simply within an “anti-inflammatory” or “pro-reparative” framework, nor should an increase in M2-like phenotypes be directly regarded as beneficial.

Animal studies have shown that exercise can influence TAM polarization states in breast cancer and lung cancer models and regulate the expression of immune checkpoint-related molecules such as programmed death-ligand 1 (PD-L1), cluster of differentiation 47 (CD47), and cluster of differentiation 24 (CD24), suggesting that exercise may participate in remodeling the tumor immune microenvironment [157,158]. In clinical studies, the PREFERABLE-EFFECT randomized controlled trial showed that supervised individualized exercise improved physical fatigue and quality of life in patients with metastatic breast cancer. The CO21 CHALLENGE study further suggested that a structured exercise program after adjuvant chemotherapy for colon cancer may improve disease-free survival outcomes [159,160]. Mechanistic studies have also shown that voluntary wheel running can suppress tumor growth through epinephrine- and IL-6-dependent NK-cell mobilization, that exercise-induced SPARC can promote tumor cell apoptosis in colon tumor models, and that a single bout of resistance training or high-intensity interval training in breast cancer survivors can increase circulating levels of Decorin, IL-6, SPARC, and OSM, with post-exercise serum inhibiting MDA-MB-231 breast cancer cell growth in vitro [74,160,161].

Overall, growing evidence supports the clinical benefits of exercise in cancer rehabilitation. However, current evidence does not yet demonstrate that exercise directly remodels TAMs and improves patient prognosis through a specific myokine. Therefore, exercise prescriptions in cancer rehabilitation should be designed with careful consideration of safety, tumor type, treatment stage, metastatic site, risk of bone metastasis, anemia, and treatment-related adverse events. Candidate myokines and immune microenvironment indicators may serve as supplementary measures for mechanistic exploration and efficacy monitoring, but they should not replace clinical outcomes such as cardiorespiratory fitness, muscle strength, physical function, cancer-related fatigue, quality of life, and disease-free or overall survival.

### 4.5. Translational Pathway: From Mechanistic Discovery to Precision Exercise Prescription and Integrated Outcome Evaluation

Overall, the translational application of exercise-induced skeletal muscle secretory networks in regulating the macrophage functional spectrum should move beyond single-mechanism explanations toward disease-contextualized exercise prescriptions, multidimensional outcome evaluation, and clear definition of evidence boundaries. Different disease scenarios require distinct translational logic. Injury rehabilitation emphasizes the temporal window of inflammatory clearance–repair transition and progressive load control. Metabolic diseases emphasize the control of chronic low-grade inflammation, improvement of insulin sensitivity, and restoration of immunometabolic homeostasis. Aging and sarcopenia emphasize the maintenance of muscle regenerative capacity, muscle strength, and functional reserve. Cancer rehabilitation emphasizes treatment tolerance, cancer-related fatigue, sarcopenia risk, and safety boundaries related to immune microenvironment modulation.

Future studies should integrate exercise-induced secretory signals, inflammatory markers, metabolic indicators, macrophage functional states, tissue repair indices, and clinical functional outcomes. Longitudinal, standardized, and time-resolved human exercise intervention studies are needed to clarify the causal relationships within this regulatory axis under different exercise modalities, doses, and recovery states.

## 5. Current Challenges and Future Perspectives

Although existing studies suggest that exercise-induced skeletal muscle secretory factors may participate in macrophage functional remodeling, regulation of the inflammatory microenvironment, and tissue repair, this field remains in a transitional stage from correlative observations to causal mechanistic validation. Much of the current evidence is still limited to observations showing that a given secretory factor increases after exercise, accompanied by changes in M1/M2-related markers. Such evidence is insufficient to demonstrate that a specific skeletal muscle-derived factor is a necessary mediator linking exercise stimulation to macrophage functional transitions. Future studies should integrate skeletal muscle-specific gene knockout, macrophage receptor blockade, neutralizing antibodies, conditional overexpression, skeletal muscle cell–macrophage co-culture, and conditioned medium transfer experiments to validate the necessity and sufficiency of candidate factors within the mechanistic chain of exercise stimulation, receptor recognition, macrophage functional remodeling, and tissue repair or metabolic improvement.

At the same time, the tissue origin and conceptual boundaries of exercise-induced secretory factors require further clarification. Not all molecules elevated after exercise can be directly defined as skeletal muscle-derived myokines. For example, FGF21, Apelin, OSM, and LIF have multiple tissue sources; BAIBA is more appropriately considered an exerkine or exercise-induced metabolite; and Sestrin mainly functions as an intracellular stress-adaptation protein. Therefore, future studies should distinguish among different categories, including myokines, exerkines, chemokines, metabolites, and intracellular stress proteins. Approaches such as skeletal muscle biopsy, arteriovenous difference measurements across muscle, skeletal muscle interstitial fluid proteomics, isotope tracing, single-cell omics, and spatial omics should be used to define the tissue origin, release kinetics, and target-cell distribution of candidate molecules.

In addition, macrophage evaluation should move beyond the traditional M1/M2 dichotomy and shift from single-marker-based classification toward functional-state analysis. In addition to phenotypic assessment, future studies should incorporate functional and metabolic indicators, including phagocytic capacity, efferocytosis, antigen presentation, cytokine profiles, mitochondrial respiration, glycolysis, fatty acid oxidation, and redox status. Given the continuing lack of human evidence, standardized, longitudinal, and time-resolved human exercise intervention studies are needed. These studies should integrate circulating secretory factors, tissue-level cellular states, inflammatory markers, and functional outcomes to construct stratified models linking exercise dose, secretory factor responses, macrophage functional states, and clinical outcomes, thereby providing a mechanistic basis for precision exercise prescription.

## 6. Conclusions

This review concludes that exercise-induced skeletal muscle secretory factors regulate macrophage function not by simply promoting an M1-to-M2 shift, but by dynamically remodeling the macrophage functional spectrum through interconnected signaling pathways, immunometabolic reprogramming, and tissue microenvironmental changes. These effects are time-dependent, tissue-specific, and disease-context-dependent, contributing to inflammation resolution, tissue repair, and metabolic homeostasis. However, stronger causal evidence from human and mechanistic studies is still needed.

## Figures and Tables

**Table 1:** Evidence hierarchy of exercise-induced skeletal muscle secretory factors in macrophage functional remodeling.

Factor	Functional Category	Human Exercise/Muscle Evidence	OCEBM Level	Macrophage-Related Evidence and Interpretation
IL-8/CXCL8	Chemotactic/recruitment	Acute exercise induces local IL-8/CXCL8-related responses in human skeletal muscle.	Level 4	Mainly regulates immune-cell recruitment through CXCR1/2; direct human macrophage evidence is lacking.
CXCL1	Chemotactic/recruitment	Evidence is mainly derived from animal studies, with multiple tissue sources.	N/A	Preclinical evidence supports CXCR2-mediated early inflammatory-cell recruitment.
CXCL12/SDF-1	Chemotactic/recruitment	Mainly supported by preclinical muscle-repair and migration studies.	N/A	Regulates CXCR4-dependent cell migration and repair-niche formation; macrophage effects are indirect.
IL-6	Immunoregulatory	Human studies demonstrate exercise-induced expression and release from contracting skeletal muscle.	Level 3	May regulate inflammatory timing and repair through IL-6R/gp130–STAT3; direct human macrophage causality remains unconfirmed.
IL-15	Immunoregulatory	Human studies and meta-analysis support acute exercise-related changes, although chronic responses are inconsistent.	Level 2 *	Macrophage effects are mainly inferred from broader immunometabolic regulation.
OSM	Immunoregulatory	Exercise-induced skeletal muscle expression is mainly supported by mouse studies.	N/A	Animal evidence supports OSMR/gp130–STAT3-mediated immune-cell recruitment and repair.
LIF	Immunoregulatory	Direct human exercise evidence is limited in the current reference set.	N/A	LIFR/gp130–STAT3 may support repair-associated macrophage programs, mainly based on mechanistic evidence.
Irisin	Metabolic reprogramming	Human exercise studies and meta-analysis support exercise-related changes in circulating irisin.	Level 2 *	Cell and animal studies support JAK2/STAT6–PPARγ/Nrf2-mediated anti-inflammatory macrophage effects.
METRNL	Metabolic reprogramming	Exercise-responsive skeletal muscle expression is supported mainly by preclinical studies.	N/A	Acts primarily through the muscle–adipose–eosinophil–macrophage network rather than direct macrophage activation.
FGF21	Metabolic reprogramming	Acute exercise can increase circulating FGF21, but skeletal muscle-specific origin is uncertain.	Level 4	Mainly regulates macrophage inflammation indirectly through FGFR1/β-Klotho, Nrf2 and NF-κB pathways.
BAIBA	Exercise-responsive metabolite	Human muscle-release evidence is insufficient; current evidence is mainly preclinical or associative.	N/A	May improve the macrophage metabolic environment through AMPK/PPARδ/Nrf2-related mechanisms.
Musclin/Ostn	Metabolic reprogramming	Exercise responsiveness and muscle origin are mainly supported by animal studies. A recent uncontrolled human study reported increased serum musclin during and after moderate-intensity aerobic exercise in 21 inactive young men; skeletal muscle-specific origin was not directly assessed.	Level 4	Preclinical evidence supports FPR2-mediated metabolic reprogramming and efferocytosis. Human macrophage evidence remains unavailable; the proposed metabolic and macrophage-related mechanisms are still based mainly on preclinical studies
Apelin	Metabolic reprogramming	Human training studies indicate increased skeletal muscle apelin expression.	Level 4	APJ-related AMPK/Akt and Nrf2/NF-κB signaling may indirectly regulate macrophage inflammation.
Myostatin	Growth/repair and matrix remodeling	A systematic review and meta-analysis of randomized studies supports resistance-training-related reductions.	Level 1	Macrophage effects are mainly indirect through regeneration, fibrosis and repair-niche remodeling.
Decorin	Growth/repair and matrix remodeling	Human myotube and acute resistance-exercise studies support muscle release.	Level 4	May influence macrophages indirectly through TGF-β/myostatin and extracellular matrix remodeling.
SPARC	Growth/repair and matrix remodeling	Acute exercise increases skeletal muscle SPARC expression, based mainly on before–after studies.	Level 4	Macrophage-related effects remain mainly preclinical and context dependent.
IGF-1	Growth/repair and matrix remodeling	Current references do not establish direct human evidence for exercise-induced muscle-derived IGF-1.	N/A	Supports regeneration through IGF-1R–PI3K/Akt/mTOR, but the cited evidence partly concerns macrophage-derived IGF-1.
VEGF-A	Growth/repair and matrix remodeling	Human exercise-related angiogenic evidence exists, but direct primary evidence in the current reference set is limited.	N/A	May indirectly influence macrophages through angiogenesis, oxygen supply and repair-niche formation.
Extracellular vesicles/miRNAs	Extracellular vesicles/non-coding RNAs	Human exercise studies report changes in EV abundance and miRNA cargo.	Level 4	Myotube-derived EVs attenuate macrophage inflammation in vitro; muscle-source tracing and in vivo targeting remain limited.

Note: Human evidence for exercise responsiveness was classified according to the 2011 Oxford Centre for Evidence-Based Medicine Levels of Evidence 2.1. The prespecified question was whether exercise alters the circulating concentration, skeletal muscle expression, or release of each factor in humans. Level 1 indicates a systematic review of randomized trials; Level 2 indicates randomized-trial evidence; Level 3 indicates a non-randomized controlled study; and Level 4 indicates uncontrolled before–after or case-series-type human evidence. “N/A” indicates that no directly relevant human exercise study was identified in the current reference set and does not indicate an absence of preclinical evidence. Evidence for macrophage regulation was assessed separately and described as direct human, human associative, animal, in vitro, or indirect mechanistic evidence. Animal and cell studies were not automatically assigned a human OCEBM level. The OCEBM level therefore reflects human exercise-response evidence rather than the certainty of the complete exercise–skeletal muscle factor–macrophage causal pathway. * The cited systematic review or network meta-analysis included heterogeneous study designs and was not restricted exclusively to randomized trials; therefore, the strongest directly relevant randomized evidence was classified as Level 2 rather than Level 1. Abbreviations: IL-8, interleukin-8; CXCL8, C-X-C motif chemokine ligand 8; CXCR1, C-X-C motif chemokine receptor 1; CXCR2, C-X-C motif chemokine receptor 2; CXCL1, C-X-C motif chemokine ligand 1; CXCL12, C-X-C motif chemokine ligand 12; SDF-1, stromal cell-derived factor 1; CXCR4, C-X-C motif chemokine receptor 4; IL-6, interleukin-6; IL-6R, interleukin-6 receptor; gp130, glycoprotein 130; STAT3, signal transducer and activator of transcription 3; IL-15, interleukin-15; OSM, oncostatin M; OSMR, oncostatin M receptor; LIF, leukemia inhibitory factor; LIFR, leukemia inhibitory factor receptor; JAK2, Janus kinase 2; STAT6, signal transducer and activator of transcription 6; PPARγ, peroxisome proliferator-activated receptor gamma; Nrf2, nuclear factor erythroid 2-related factor 2; METRNL, meteorin-like protein; FGF21, fibroblast growth factor 21; FGFR1, fibroblast growth factor receptor 1; NF-κB, nuclear factor kappa B; BAIBA, β-aminoisobutyric acid; AMPK, AMP-activated protein kinase; PPARδ, peroxisome proliferator-activated receptor delta; FPR2, formyl peptide receptor 2; APJ, apelin receptor; Akt, protein kinase B; TGF-β, transforming growth factor beta; IGF-1, insulin-like growth factor 1; IGF-1R, insulin-like growth factor 1 receptor; PI3K, phosphoinositide 3-kinase; mTOR, mechanistic target of rapamycin; VEGF-A, vascular endothelial growth factor A; EVs, extracellular vesicles; miRNAs, microRNAs; OCEBM, Oxford Centre for Evidence-Based Medicine; N/A, not applicable.

## Data Availability

No new data were created or analyzed in this study. Data sharing is not applicable to this article.

## Abbreviations

The following abbreviations are used in this manuscript:

ActRIIB	Activin receptor type IIB
Akt	Protein kinase B
ALK4/5	Activin receptor-like kinase 4/5
AMPK	AMP-activated protein kinase
AMPKα1	AMP-activated protein kinase alpha 1
ANP	Atrial natriuretic peptide
APJ	Apelin receptor
Arg1	Arginase 1
AWGS	Asian Working Group for Sarcopenia
BAIBA	β-aminoisobutyric acid
BDNF	Brain-derived neurotrophic factor
CCL2	C-C motif chemokine ligand 2
CCR2	C-C motif chemokine receptor 2
CD8	Cluster of differentiation 8
CD24	Cluster of differentiation 24
CD44	Cluster of differentiation 44
CD47	Cluster of differentiation 47
CD86	Cluster of differentiation 86
CD206	Cluster of differentiation 206
cGMP	Cyclic guanosine monophosphate
COX-2	Cyclooxygenase-2
CRP	C-reactive protein
CXCL1	C-X-C motif chemokine ligand 1
CXCL8	C-X-C motif chemokine ligand 8
CXCL12	C-X-C motif chemokine ligand 12
CXCR1	C-X-C motif chemokine receptor 1
CXCR2	C-X-C motif chemokine receptor 2
CXCR4	C-X-C motif chemokine receptor 4
ECAR	Extracellular acidification rate
ECM	Extracellular matrix
EVs	Extracellular vesicles
FGF21	Fibroblast growth factor 21
FGFR1	Fibroblast growth factor receptor 1
FNDC5	Fibronectin type III domain-containing protein 5
FPR2	Formyl peptide receptor 2
GDF-8	Growth differentiation factor 8
gp130	Glycoprotein 130
GPR109A	G protein-coupled receptor 109A
HO-1	Heme oxygenase-1
ICAM-1	Intercellular adhesion molecule 1
IGF-1	Insulin-like growth factor 1
IGF-1R	Insulin-like growth factor 1 receptor
IKK	IκB kinase
IL-1β	Interleukin-1β
IL-4	Interleukin-4
IL-4Rα	Interleukin-4 receptor alpha
IL-6	Interleukin-6
IL-6R	Interleukin-6 receptor
IL-8	Interleukin-8
IL-10	Interleukin-10
IL-13	Interleukin-13
IL-15	Interleukin-15
IL-41	Interleukin-41
iNOS	Inducible nitric oxide synthase
IRAK4	Interleukin-1 receptor-associated kinase 4
IRF3	Interferon regulatory factor 3
IRG1	Immune-responsive gene 1
IκB	Inhibitor of κB
JAK	Janus kinase
JAK1	Janus kinase 1
JAK2	Janus kinase 2
LIF	Leukemia inhibitory factor
LIFR	Leukemia inhibitory factor receptor
LPS	Lipopolysaccharide
M1	Classically activated pro-inflammatory macrophage phenotype
M2	Alternatively activated reparative macrophage phenotype
MAPK	Mitogen-activated protein kinase
MCP-1	Monocyte chemoattractant protein 1
MDA-MB-231	Human breast cancer cell line MDA-MB-231
METRNL	Meteorin-like protein
miRNA	MicroRNA
miRNAs	MicroRNAs
Mrc1	Mannose receptor C-type 1
MRGPRD	MAS-related G protein-coupled receptor D
mTOR	Mechanistic target of rapamycin
MyD88	Myeloid differentiation primary response 88
N/A	Not applicable
NAD^+^	Nicotinamide adenine dinucleotide
NF-κB	Nuclear factor kappa B
NK	Natural killer
NPR-C	Natriuretic peptide receptor C
NQO1	NAD(P)H quinone oxidoreductase 1
Nrf2	Nuclear factor erythroid 2-related factor 2
OCEBM	Oxford Centre for Evidence-Based Medicine
OCR	Oxygen consumption rate
OSM	Oncostatin M
OSMR	Oncostatin M receptor
Ostn	Osteocrin
p-Smad2/3	Phosphorylated SMAD family member 2/3
PD-L1	Programmed death-ligand 1
PGC-1α	Peroxisome proliferator-activated receptor gamma coactivator 1-alpha
PGC-1β	Peroxisome proliferator-activated receptor gamma coactivator 1-beta
PI3K	Phosphoinositide 3-kinase
PPARα	Peroxisome proliferator-activated receptor alpha
PPARδ	Peroxisome proliferator-activated receptor delta
PPARγ	Peroxisome proliferator-activated receptor gamma
RNA	Ribonucleic acid
ROS	Reactive oxygen species
SDF-1	Stromal cell-derived factor 1
sIL-6R	Soluble interleukin-6 receptor
Smad2/3	SMAD family member 2/3
SOCS3	Suppressor of cytokine signaling 3
SPARC	Secreted protein acidic and rich in cysteine
STAT	Signal transducer and activator of transcription
STAT1	Signal transducer and activator of transcription 1
STAT3	Signal transducer and activator of transcription 3
STAT6	Signal transducer and activator of transcription 6
TAMs	Tumor-associated macrophages
TBK1	TANK-binding kinase 1
TGF-β	Transforming growth factor beta
TLR	Toll-like receptor
TLR4	Toll-like receptor 4
TNF-α	Tumor necrosis factor alpha
TRAF6	Tumor necrosis factor receptor-associated factor 6
TRIF	TIR-domain-containing adapter-inducing interferon-β
UCP1	Uncoupling protein 1
VEGF	Vascular endothelial growth factor
VEGF-A	Vascular endothelial growth factor A
VEGFR	Vascular endothelial growth factor receptor
VEGFR-2	Vascular endothelial growth factor receptor 2
YAP	Yes-associated protein
β-Klotho	Beta-Klotho
